# Fast formation and growth of high-density Sn whiskers in Mg/Sn-based solder/Mg joints by ultrasonic-assisted soldering: Phenomena, mechanism and prevention

**DOI:** 10.1038/srep27522

**Published:** 2016-06-08

**Authors:** M. Y. Li, H. F. Yang, Z. H. Zhang, J. H. Gu, S. H. Yang

**Affiliations:** 1 State Key Laboratory of Advanced Welding and Joining, Harbin Institute of Technology, Harbin, 150001, China; 2 Fujian Key Laboratory of Advanced Materials, Department of Materials Science and Engineering, College of materials, Xiamen University, Xiamen, 361005, China; 3 Shanghai Aerospace Equipments Manufacturer, Shanghai, 200245, China

## Abstract

A universally applicable method for promoting the fast formation and growth of high-density Sn whiskers on solders was developed by fabricating Mg/Sn-based solder/Mg joints using ultrasonic-assisted soldering at 250 °C for 6 s and then subjected to thermal aging at 25 °C for 7 d. The results showed that the use of the ultrasonic-assisted soldering could produce the supersaturated dissolution of Mg in the liquid Sn and lead to the existence of two forms of Mg in Sn after solidification. Moreover, the formation and growth of the high-density whiskers were facilitated by the specific contributions of both of the Mg forms in the solid Sn. Specifically, interstitial Mg can provide the persistent driving force for Sn whisker growth, whereas the Mg_2_Sn phase can increase the formation probability of Sn whiskers. In addition, we presented that the formation and growth of Sn whiskers in the Sn-based solders can be significantly restricted by a small amount of Zn addition (≥3 wt.%), and the prevention mechanisms are attributed to the segregation of Zn atoms at grain or phase boundaries and the formation of the lamellar-type Zn-rich structures in the solder.

Spontaneous Sn whisker growth, which causes short circuits, is one of the most serious reliability issues in virtually all microelectronic devices^1^,^2^,^3^. However, the uncertainties associated with the formation of these whiskers greatly complicate the attempts to predict if or when they may emerge^4^. Many scholars believe that the major challenge in achieving an in-depth understanding and further prevention of Sn whisker formation is the development of an effective accelerated detection method. However, no industry-accepted accelerated test method has been developed that can effectively evaluate the propensity of a particular solder to form Sn whiskers^5^,^6^. Therefore, the elimination of the whisker risks in varied Sn-based solders warrants further research.

The design of an accelerated detection method is primarily hindered by the low temperature range (*i.e.*, 25–60 °C) in which Sn whisker growth has been reported to occur^4^. When the temperature is less than 25 °C, the kinetics of the Sn whisker growth may be sluggish because of slow Sn self-diffusion; when the temperature exceeds 60 °C, the local compressive stresses that drive the whisker growth will be insufficient because of the fast creep relief of the Sn lattices. Accordingly, typical experimental observations of Sn whisker growth in solders have always been conducted on very long time scales, even over the course of several years^7^. Although Suganuma *et al.*^8^ has demonstrated that the Sn whiskers can be formed at relatively high temperatures when the compressive stress in the solder is sufficiently high, the actual evaluation period still endured for months. However, the study by Suganuma *et al.* provided important insights into the development of an optimal accelerated detection method at ambient temperature, which should provide large and long-range compressive stresses in solders.

Chiu and Lin^9^ and Choi *et al.*^10^ proposed that the origin of the compressive stresses that would promote Sn whisker growth could be mechanical, thermal and chemical. Mechanical and thermal stresses were finite and were not sustainable; however, the chemical stress that arose from the formation of the intermetallic compounds (IMCs) would be fast and sustained. Therefore, the methodology that can promote IMC formation in various solders becomes critical. Generally, the premixed additions in solders (*e.g.*, metal particles^11^,^12^ or rare-earth elements)^13^,^14^ are considered to dramatically increase the sources of the elements that are supplied for IMC formation. However, these additions will also change the original solder composition, which may disrupt the propensity of a particular solder to form Sn whiskers. In addition, because the IMC formation is a diffusion-controlled reaction^12^, the newly generated IMC layer may become an atomic diffusion barrier that blocks the continuous IMC growth over time^15^. Hence, the mixing of additions in solders may create some inevitable problems.

Notably, Mg can react with Sn to form the Mg_2_Sn phase; however, the solubility of Mg in Sn at room temperature is low (0.49–0.97 at%)^16^. Moreover, Mg will not react with other elements (*e.g.,* Cu, Ag, Zn, Pb) below 250 °C^17^. Hence, if Mg is used in Sn-based solders as the metal addition, then it may have only a slight influence on the solder composition, especially in the case of some novel composite Sn-based solders^18^. Additionally, based on our previous study^19^, when the ultrasonic-assisted soldering (UAS) was applied to Cu/Sn/Cu sandwich joints, the ultrasonic energy that created the shock waves, liquid-solder micro-jets and localized high temperatures can accelerate the dissolution of the Cu atoms to produce a supersaturation of Cu atoms in the liquid Sn, resulting in a large IMC formation after solder solidification. Thus, UAS may be a simple and convenient method to accelerate elemental dissolution to form IMCs and to promote massive compressive stresses in solders. In this study, a universally applicable method using UAS to promote the dissolution of Mg in varied Sn-based solders was developed to estimate the solder propensities of the Sn whisker formation and growth. The reasons for the fast formation and growth of high-density Sn whiskers in solders were analyzed, and the methods and mechanisms for suppressing the Sn whisker risks were also proposed.

## Results and Discussion

### Microstructures of the Mg/Sn/Mg joints after UAS

**Figure 1:**
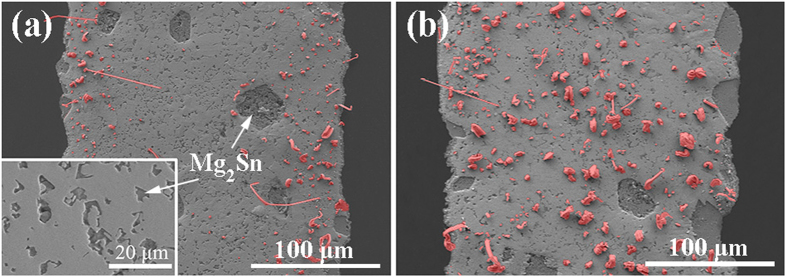
Microstructures of the two Mg/Sn/Mg joints after the UAS at 250 °C for a short period followed by thermal aging at 25 °C for 7 d: (**a**) after UAS for 3 s; (**b**) after UAS for 6 s. Note that the Sn whiskers generated in the Sn fillers are highlighted in red.

Figure 1 shows the microstructures of two Mg/Sn/Mg joints after UAS at 250 °C for 3–6 s followed by thermal aging at 25 °C for 7 d. In the case of UAS of 3 s in Fig. 1a, both of the Mg sheets were wetted well with the Sn filler, despite the irregular contours that appeared at both of the joint interfaces. Moreover, based on the Mg-Sn phase diagram^16^ and energy dispersive spectroscopy (EDS) analysis, we determined that the Mg_2_Sn phase was formed at the joint interfaces and in the Sn matrix. Most of the Mg_2_Sn grains exhibited fine seaweed-type shapes and were 1–6 μm in size, whereas very few of the grains have massive shapes and were 20–30 μm in size. Interestingly, a large number of Sn protuberances (*i.e.*, the initial whiskers) were observed in the Sn matrix near both of the Mg/Sn interfaces, and the roots of these whiskers were always located at the Mg_2_Sn/Sn interfaces (for more details, see Section A of the Supplementary material). Although the average length of these whiskers was less than 10 μm, the density of the whiskers per unit area reached 3.96 × 10^9^ number/m^2^, which was more than one order of magnitude larger than the highest value reported in Sun’s study^20^. In the case of UAS of 6 s in Fig. 1b, the Mg sheets were also wetted well with the Sn filler, and two types of Mg_2_Sn grains with different shapes were also observed. In this case, a larger number of Sn whiskers were generated, and they were evenly distributed in the Sn matrix. The average height of these Sn whiskers was in the range of 10–20 μm, and the density of the whiskers per unit area approached 6.95 × 10^9^ number/m^2^, which was 1.75 times larger than that of the whiskers in Fig. 1a.

We developed an effective method to produce the high-density Sn whiskers in Mg/Sn/Mg joints via UAS and short-duration aging. However, the formation and growth mechanisms of these Sn whiskers were unclear. Considering our experimental configuration and procedure, the corresponding mechanisms might be related to the Mg_2_Sn phase. We observed that the average widths of the soldering seams after UAS of 3 and 6 s were 215.77 and 227.91 μm, respectively; these seams were wider than the 200 μm width that was limited by the artificial spacers. The increased seam widths were obviously due to the continuous dissolution of Mg sheets, which was a consequence of ultrasonic effects; accordingly, we estimated that the ideal concentrations of the Mg atoms in these two fillers should be 8.34 at% and 13.97 at%, respectively. Meanwhile, from the EDS analysis results, the actual Mg concentrations in Fig. 1a,b were 6.94 at% and 12.38 at%, respectively. Although the ideal and actual concentrations are not identical, they are similar; furthermore, they are at least one order of magnitude larger than the solid solubility of Mg in the solid Sn at 25 °C (0.49–0.97 at%)^16^. The ultrasonic energy can promote the dissolution of Mg in the liquid Sn, especially in the case of the joint subjected to UAS for 6 s. However, the characteristics of the dissolved Mg atoms in the Sn fillers after solder solidification remained unclear. According to the images of the Mg_2_Sn areas in Fig. 1a,b, the calculated concentrations of Mg in the Sn were 5.55 and 6.84 at%, respectively (for more details, see Section B of the Supplementary material). The large differences between the actual (or ideal) concentrations and the calculated concentrations indicated that the dissolved Mg atoms in the liquid Sn may not completely transform into the Mg_2_Sn phase after solder solidification. Thus, some Mg atoms in the solid Sn may exist in a non-Mg_2_Sn form.

### Identification of interstitial Mg in solid Sn

**Figure 2:**
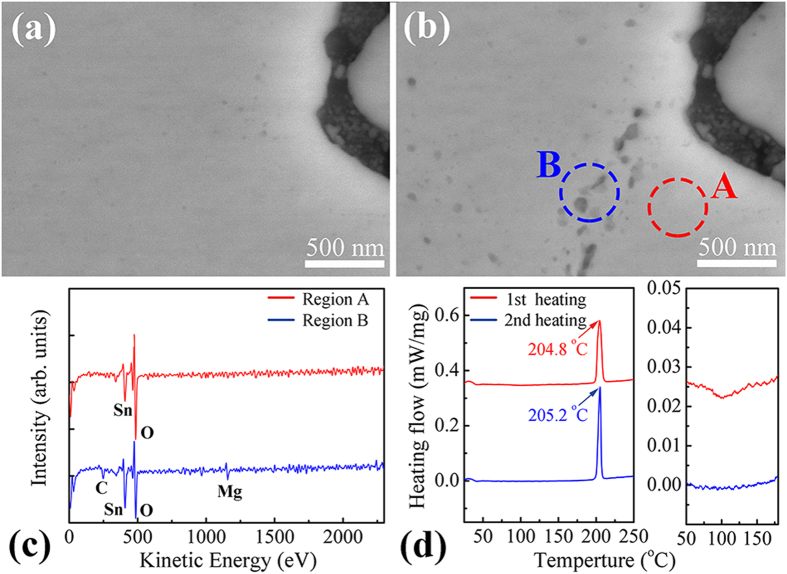
*In situ* observation of the surface evolution for one Mg/Sn/Mg sandwich joint after UAS at 250 °C for 6 s followed by thermal aging at 25 °C for (**a**) 0 d and (**b**) 7 d. (**c**) AES spectra of Region A and Region B in Fig. 3b. (d) Two DSC heat-flow curves of one particular solder. These curves were acquired in the ranges of 25–250 °C and 50–200 °C for the as-fabricated Mg/Sn/Mg joint subjected to UAS at 250 °C for 6 s.

Figure 2a,b show the SEM images of the *in situ* observation of the surface evolution for a Mg/Sn/Mg joint after UAS at 250 °C for 6 s followed by thermal aging at 25 °C for 0 and 7 d, respectively. Although no Sn whisker growth phenomenon was observed, several distinct gray particles (30–80 nm in diameter) generated in the solder surface after thermal aging of 7 d were evident. Interestingly, as shown in the AES spectra in Fig. 2c, a relatively intense Mg signal at 1146 eV was detected in Region B of Fig. 2b but was not detected in Region A. Thus, these gray particles in Region B should be the Mg_2_Sn phase. To generate these Mg_2_Sn particles, the form of Mg in the solid Sn should be carefully considered. If the Mg in the Sn was entirely in the form of the Mg_2_Sn phase, then the formation of these gray particles should involve Ostwald ripening^21^. In this case, the small Mg_2_Sn particles would shrink while the larger ones would grow; however, no volumetric change could occur in the solder to produce compressive stresses. Another possibility was that a portion of Mg in the solid Sn would react with Sn to form the Mg_2_Sn phase, and the other portion would disperse into the Sn lattices as interstitial atoms. Because the interstitial Mg atoms in the solid Sn lattices do not exhibit thermodynamic stability, they would react with the Sn atoms over time. Because the molar volumes of the Sn phase and Mg_2_Sn phase were 1.61 × 10^−5^ and 4.74 × 10^−5^ m^3^/mol, respectively, the formation of the Mg_2_Sn phase could result in a 194.4% expansion in the volume and might become a source of large compressive stresses in the solid Sn.

To identify the true situation, a DSC test, involving the particular solder derived from an Mg/Sn/Mg joint after UAS at 250 °C for 6 s, was performed under Ar atmosphere and was cycled twice in the range of 25–250 °C. Based on the DSC heat-flow curve of the first heating process (red line in Fig. 2d), an endothermic peak with an enthalpy of 82.03 J/g was detected at 204.8 °C. According to the previous study^16^, the formation of this endothermic peak was related to Mg_2_Sn dissolution in the liquid solder, and the peak temperature above 203.5 °C indicated that the test solder was hypereutectic with respect to the Mg content (>9.6 at%). In contrast, in the second heating process (blue line in Fig. 2d), the endothermic peak shifted to 205.2 °C, and the corresponding enthalpy increased to 82.21 J/g. These increases in endothermic peak temperature and endothermic enthalpy clearly demonstrated that the content of Mg_2_Sn in the Sn increased during the thermal history. Therefore, the interstitial Mg atoms were present in the as-fabricated Sn matrix after solder solidification. More importantly, based on the DSC heat-flow curves for the temperature range from 50–200 °C, a broad exothermic peak was observed during the first heating process but was absent in the second heating process. This abnormal peak was most likely directly related to the exothermic formation reaction of the Mg_2_Sn phase, and its disappearance suggested that the Mg atoms in the Sn after the first heating-cooling cycle have completely transformed into the Mg_2_Sn phase. Accordingly, we demonstrated that the Mg atoms in the solid Sn after UAS could exist as interstitial atoms rather than entirely as the Mg_2_Sn phase.

### Influence of interstitial Mg on whisker growth

Because the interstitial Mg atoms will inevitably transform into the Mg_2_Sn phase at 25 °C over time, the strain energy change of the solder (Δ*E*_*S*_) that is due to the Mg_2_Sn formation should be as follows (for additional details, see Section C of the Supplementary material):


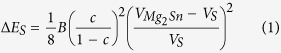


where *B* is the bulk modulus of a particular solder, *c* is the atomic concentration of the interstitial Mg in Sn, and *V*_Mg2Sn_ and *V*_S_ are the molar volumes of the Mg_2_Sn and Sn phases, respectively. Thus, the driving force for the Sn whisker growth in the solder would be determined by the solder bulk modulus and the interstitial Mg concentration.

**Figure 3:**
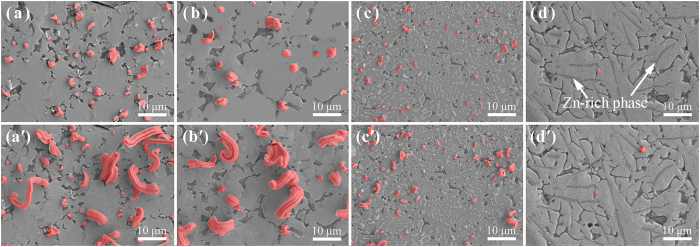
*In situ* observations of the surface evolutions for four types of Mg/Sn-based solder/Mg sandwich joints subjected to UAS at 250 °C for 6 s followed by thermal aging at 25 °C: (**a**) and (a’) SnAg_3.5_ aged for 0 and 7 d, respectively; (**b**) and (b’) SnCu_0.7_ aged for 0 and 7 d, respectively; (**c**) and (c’) SnPb_37_ aged for 0 and 7 d, respectively; (**d**) and (d’) SnZn_9_ aged for 0 and 7 d, respectively. Note that the Sn whiskers generated in the Sn fillers are highlighted in red.

Figure 3 shows the *in situ* observations of the surface evolution for four types of the Mg/Sn-based solder/Mg joints (*i.e.*, SnAg_3.5_, SnCu_0.7_, SnPb_37_, and SnZn_9_) after UAS at 250 °C for 6 s followed by thermal aging at 25 °C for 0 and 7 d, respectively. Based on the EDS analysis, the Mg concentrations in these solders were determined to be 13.62, 13.37, 10.68 and 12.03 at%, respectively. Because the amounts of the Mg_2_Sn phase that formed during solidification in varied solders should be virtually constant, the concentrations of the interstitial Mg atoms in these solid solders can be considered nearly identical. Moreover, according to previous studies^4^,^22^−^24^, Young’s modulus for the SnAg_3.5_, SnCu_0.7_, SnPb_37_, and SnZn_9_ solders was 50, 42.5, 30, and 48.5 GPa, respectively. Using these data in Eq. 1 (because the bulk modulus and Young’s modulus are related, for convenience we use Young’s modulus here), the driving forces that promote the Sn whisker growth in the solders can be predicted to increase in the order of Δ*E*_*S*_(SnAg_3.5_) > Δ*E*_*S*_(SnZn_9_) > Δ*E*_*S*_(SnCu_0.7_) > Δ*E*_*S*_ (SnPb_37_). However, by calculating from Fig. 3 (the detailed data are listed in Table 1), the volume of the Sn whiskers per unit area (Δ*V*_S_ = *l*·*ρ*) from 0–7 d increased in the order of Δ*V*_S_(SnAg_3.5_) > Δ*V*_S_(SnCu_0.7_) > Δ*V*_S_(SnPb_37_) ≫ Δ*V*_S_(SnZn_9_). Hence, the growth rate of the Sn whiskers was related to the driving force inside the solder, and this result is consistent with previous findings^1^,^2^,^13^,^25^,^26^.

Given that the risk of the Sn whisker growth was primarily caused by bridging between the adjacent joints because of its fast and large growth^25^,^26^, the existence of certain individual whiskers, whose growth lengths were beyond 5 μm, could be very dangerous. Therefore, the propensities of the Sn whisker growth in the SnAg_3.5_, SnCu_0.7_, and SnPb_37_ solders would be evaluated as “High-risk”, “High-risk”, and “Low-risk”, respectively. Unexpectedly, although the driving force inside the SnZn_9_ solder was large compared with those in the SnCu_0.7_ and SnPb_37_ solders, the whisker growth volume in SnZn_9_ was nearly two orders of magnitude less than that in the other solders (Table 1). More importantly, the whisker growth lengths in the SnZn_9_ solder after thermal aging of 7 d were less than 0.8 μm. Because the SnZn_9_ solder appeared to significantly inhibit the Sn whisker growth, we believed that the risk of growing Sn whiskers in this solder should be very low.

### Influence of the Mg_2_Sn phase on whisker formation

Another issue deserving our attention was the evaluation of the risk of forming Sn whiskers in varied solders. As the number of Sn whiskers produced in a unit area of the solder surface increased, the greater the probability of forming long whiskers. Chason *et al.*^27^ concluded that the Sn whiskers only grew from a small number of surface sites; thus, there were certain features about these sites that caused whiskers to preferentially initiate there, *e.g.*, suitable grain orientations^10^,^14^,^26^, weak oxide layers^8^,^28^, surface defects^28^,^29^, and IMC formation^13^,^14^. Following these findings, a Sn whisker would be prone to emerge at the interface (*e.g.*, the two-phase interface and the grain boundary), because numerous surface defects, broken oxides and IMCs would likely cluster nearby. Therefore, although the formation of a Sn whisker is a probability event^25^, we may increase the formation probability of the Sn whiskers when the interfacial length in the solder could be increased.

Using UAS, the dissolution of the Mg atoms could be significantly enhanced in the liquid Sn; thus, numerous seaweed-type Mg_2_Sn grains would be scattered throughout the Sn matrix after solder solidification (as shown in Figs 1 and 3), and the resultant Mg_2_Sn/Sn interfaces in the solders could promote the high-density whisker formation, regardless of the solder components. Based on Fig. 3 and Table 1, high-density Sn whiskers were observed on the solder surfaces, and the formation densities of the whiskers per unit area (*ρ*) in the solders after thermal aging of 0 d (or 7 d) increased in the order of *ρ*_*0*_(SnPb_37_) > *ρ*_*0*_(SnAg_3.5_) > *ρ*_*0*_(SnCu_0.7_). Meanwhile, the interfacial lengths (*L*) in the solders, which were measured via the digital image method described in the Supplementary material, increased in the order of *L*(SnPb_37_) > *L*(SnAg_3.5_) > *L*(SnCu_0.7_). These results showed that the formation of the Sn whiskers was directly correlated with the interfacial length in the solder. Moreover, the formation density of the Sn whiskers in the SnPb_37_ solder was more than two times larger than that in the SnAg_3.5_ or SnCu_0.7_ solder after thermal aging of 0 or 7 d. The possible reason for this result was that in addition to the Mg_2_Sn/Sn phase interfaces, there were numerous Sn-rich/Pb-rich phase interfaces inside of the SnPb_37_ solder. Hence, the propensities of forming Sn whiskers in the SnAg_3.5_, SnCu_0.7_, and SnPb_37_ solders were evaluated as “Moderate-risk”, “Moderate-risk”, and “High-risk”, respectively. In addition, we found that the formation quantity of the Sn whiskers in the SnZn_9_ solder was very limited, far less than one order of magnitude compared with those in the other solders. Therefore, the propensity of forming Sn whiskers in this solder should be “Ultralow-risk”, and the reason will be discussed later.

### Influence of interstitial Mg on solder matrix

**Figure 4:**
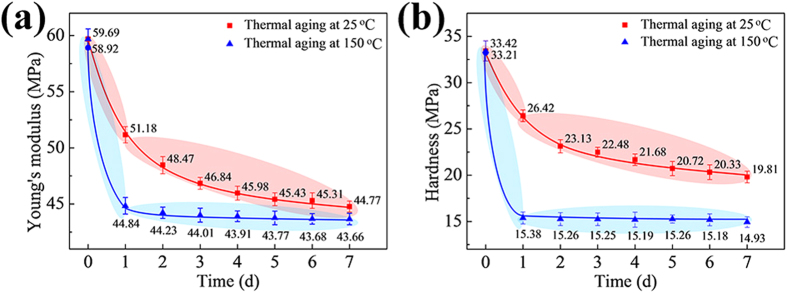
Evolutional curves of the mechanical properties of the solder matrixes for two as-fabricated Mg/SnCu_0.7_/Mg joints after thermal aging at 25 and 150 °C for 0–7 d, respectively: (**a**) Young’s modulus; (**b**) Hardness. Note that the data were obtained by instrumented nanoindentation tests.

Figure 4a shows Young’s modulus (YM) of the solder matrixes in two as-fabricated Mg/SnCu_0.7_/Mg joints after thermal aging at 25 and 150 °C for 0–7 d, respectively. For 0 d, the YMs of the two joints were 59.69 MPa for thermal aging at 25 °C (red line in Fig. 4a) and 58.92 MPa for thermal aging at 150 °C (blue line in Fig. 4a), respectively; both of these results were much higher than that reported in the reference (42.5 GPa)^24^. However, as the thermal aging time increased, both of the evolutional curves showed a tendency to rapidly decrease from 0–1 d and then slowly decrease from 2–7 d. Specifically, the YM after thermal aging at 25 °C for 1 d was 51.18 MPa, which was 85.7% of the initial value, while the YM after thermal aging at 25 °C for 7 d was 44.77 MPa, which was 75.0% of the initial value. The decrease in the YM for the first day was 1.33 times larger than that for the subsequent 6 days. Moreover, the YMs after thermal aging at 150 °C for 1 and 7 d were 44.84 and 43.66 MPa, respectively, indicating that a 97.4% decrease in YM occurred in the first day.

Figure 4b shows the hardness (HD) of two as-fabricated Mg/SnCu_0.7_/Mg joints after thermal aging at 25 and 150 °C for 0–7 d, respectively. For 0 d, the HDs of the two joints were 33.42 MPa for thermal aging at 25 °C (red line in Fig. 4b) and 33.21 MPa for thermal aging at 150 °C (blue line in Fig. 4b), respectively. As the thermal aging time increased, the evolutional curves of HDs showed a similar tendency to that in Fig. 4a. Specifically, the HD after thermal aging at 25 °C for 1 d was 26.42 MPa, which was 79.1% of the initial value, while the HD after thermal aging at 25 °C for 7 d was 19.81 MPa, which was 59.3% of the initial value. The decreasing value of the HD for the first day was 1.06 times larger than that for the subsequent 6 days. More obviously, the HDs after thermal aging at 150 °C for 1 and 7 d were 15.38 and 14.93 MPa, respectively, indicating that a 97.5% decrease in HD occurred during the first day.

The increased YMs and HDs of the solder matrixes for 0 d may depend on two interstitial-Mg-related strengthening mechanisms (*i.e.*, the solid solution strengthening and precipitation hardening) due to the reduction of the dislocation mobility^30^. As demonstrated, there were numerous interstitial Mg atoms existing in the solid Sn after UAS. Therefore, the interstitial Mg atoms would cause the Sn lattice distortions that impeded the dislocation motion, increasing the mechanical strengths of the solder matrixes. However, the interstitial Mg atoms were not stable, and they were prone to thermodynamically transform to the Mg_2_Sn phase and precipitate in the solder matrix (Fig. 2a,b). Because the YD and HD values of the Mg_2_Sn phase were 74.78 and 1.2 GPa, respectively^31^,^32^, the precipitated Mg_2_Sn particles would act as pining points to reduce dislocation mobility, thereby increasing the mechanical strengths of the solder matrixes.

When the thermal aging time increased, the interstitial Mg atoms would significantly decrease in the solid Sn due to phase transformation, while the small precipitated Mg_2_Sn particles would also shrink and disappear due to Ostwald ripening^21^. Therefore, the YMs and HDs of the solder matrixes would definitely decrease over time from 1–7 d. Moreover, the rates of the phase transformation and the atomic diffusion were temperature-dependent^12^; thus, the losses of YM and HD for the solder matrix after thermal aging at 150 °C would be much faster than those for the solder matrix after thermal aging at 25 °C. In addition, because the losses of the mechanical strengths of the samples for the first day would exceed 50% of the total losses for 0–7 d, we may further reduce the evaluation time of the propensity of Sn whisker risk in varied solders (*e.g.*, the evaluation time can be reduced to 1 d).

### Prevention of Sn whisker risks via Zn addition

**Figure 5:**
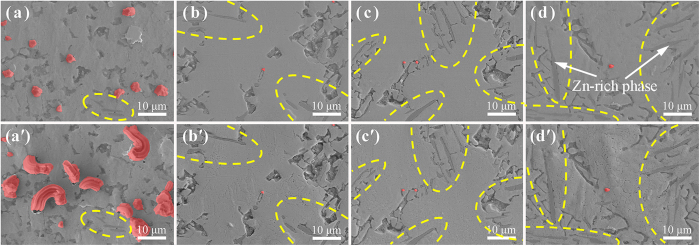
*In situ* observations of the surface evolutions for four types of Mg/SnCu_0.7_Zn_x_/Mg sandwich joints after UAS at 250 °C for 6 s followed by thermal aging at 25 °C: (**a**) and (a’) Mg/SnCu_0.7_Zn_1_/Mg aged for 0 and 7 d, respectively; (**b**) and (b’) Mg/SnCu_0.7_Zn_3_/Mg aged for 0 and 7 d, respectively; (**c**) and (c’) Mg/SnCu_0.7_Zn_5_/Mg aged for 0 and 7 d, respectively; (**d**) and (d’) Mg/SnCu_0.7_Zn_7_/Mg aged for 0 and 7 d, respectively. In addition, the clusters of the Zn-rich phase are marked by yellow dotted lines.

The most challenging task in evaluating the Sn whisker issues is not to determine the solder propensity for whisker growth but to find an effective method to prevent the spontaneous whisker formation and growth in varied solders. Based on our mentioned results, the restriction of Sn whisker risks in the SnZn_9_ solder attracted our attention. Figure 5 shows the *in situ* observations of the surface evolutions of four types of Mg/SnCu_0.7_Zn_x_/Mg joints (*i.e.*, Mg/SnCu_0.7_Zn_1_/Mg, Mg/SnCu_0.7_Zn_3_/Mg, Mg/SnCu_0.7_ Zn_5_/Mg, and Mg/SnCu_0.7_Zn_7_/Mg) after UAS at 250 °C for 6 s followed by thermal aging at 25 °C for 0 and 7 d. Compared with Fig. 3b,d, the clusters of the Zn-rich phase (marked by yellow dotted lines in Fig. 5) were clearly observed on the surfaces of all the SnCu_0.7_Zn_x_ solders. Moreover, the particle of the Zn-rich phase was lamellar-type, and its density and length increased as the Zn addition increased. More importantly, the formation densities and growth rates of the Sn whiskers in the SnCu_0.7_Zn_x_ solders remarkably decreased with increasing Zn addition, especially when its mass concentration was more than 3 wt.%; only one or two Sn whiskers could be found at the Mg_2_Sn/Sn interface distant from the Zn-rich clusters. Additionally, the experiments of the SnAg_3.5_ solder with Zn addition were also performed, and we obtained similar results. Therefore, we believed that the Sn whisker risks in the Sn-based solders could be significantly restricted by a small amount of Zn addition (≥3 wt.%).

**Figure 6:**
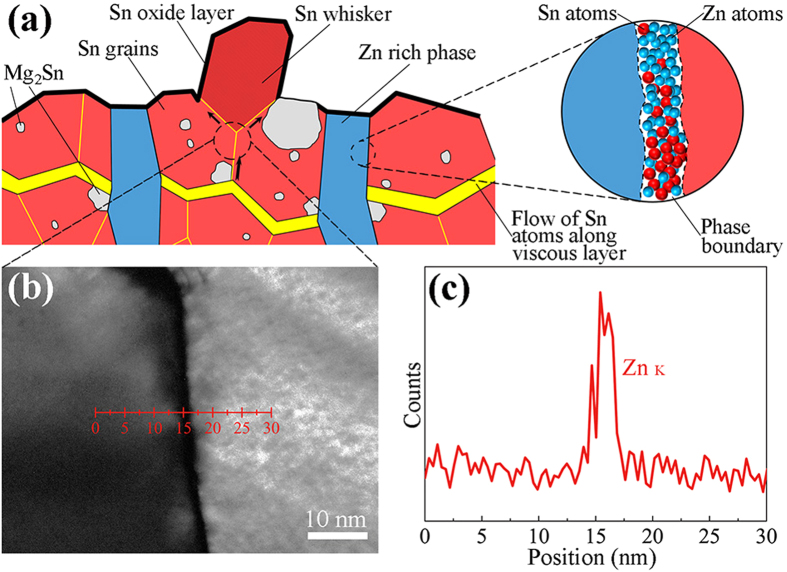
(**a**) Schematic illustration of the interface fluid flow in the SnZn_9_ solder; (**b**) Locally magnified TEM image of two adjacent Sn grains located beneath a Sn whisker. The sample was fabricated in a Mg/SnZn_9_/Mg sandwich joint after UAS at 250 °C for 6 s followed by thermal aging at 25 °C for 0 d; (**c**) Zn distribution along the line in a part of the scanned image using TEM.

The prevention mechanism of the Sn whisker risks after Zn addition has not yet been determined. According to the fluid flow theory proposed by Howard^2^, there was an anomalously fast solid-state mass transport layer beneath the Sn grains; the Sn atoms could constantly migrate along this viscous layer and preferentially flow towards the local low-stress regions. Following this mechanism, we assumed in Fig. 6a that there was also a viscous layer beneath the Sn grains; however, this viscous layer was blocked by the broken lamellar-type structures of the Zn-rich phase (*i.e.*, the eutectic structures of the Sn-Zn alloys)^33^. Therefore, it was difficult to push a Sn grain out from its base to nucleate a whisker because the flow of the Sn atoms cannot be sustained along the viscous layer to reach the region beneath the grain. Thus, we believed that the long-distance self-diffusion of the Sn atoms in the solid Sn would be restrained by the lamellar-type structures of the Zn-rich phase.

Figure 6b shows a TEM image of two adjacent grains located beneath a Sn whisker, and the sample was fabricated in a Mg/SnZn_9_/Mg sandwich joint after UAS at 250 °C for 6 s followed by thermal aging at 25 °C for 0 d. Via the TEM-EDS analysis of Fig. 6c, an obvious increase in the Zn content (4.78 at%) was detected at the grain boundary between these two Sn grains. Because the solubility of Zn in the solid Sn at 25 °C is less than 0.6 at%^34^, the segregation of the Zn atoms in the grain boundary would generate an enhanced steric hindrance effect that blocked the short-distance self-diffusion of the Sn atoms in the solid Sn. Therefore, although there might be a flow of the Sn atoms along the fragment of the viscous layer and although there were sufficient compressive stresses due to the phase transformation of the interstitial Mg to Mg_2_Sn, it is difficult to extrude a Sn whisker without a supply of Sn atoms because of the steric hindrance of the Zn atoms at the Sn grain boundaries or the Mg_2_Sn/Sn phase boundaries. In addition, due to the larger amount of the Zn atoms close to the Zn-rich phase, Sn whiskers would be even less likely to nucleate near the clusters of the Zn-rich phase. Accordingly, we believed that if the Sn diffusion in the solders can be suppressed by the addition of some barrier atoms (*e.g.*, Zn atoms) or the formation of some lamellar structures to block the viscous layer, then the formation and growth of the Sn whiskers might become controllable.

In summary, a universally applicable method to evaluate the solder propensity for Sn whisker formation was investigated in Mg/Sn-based solder/Mg sandwich joints via UAS and short-duration aging. Our results showed that the Mg atoms in the as-fabricated solders existed in two forms that have different effects. The Mg_2_Sn form can increase the phase interfacial length in the solder and promote the formation density of the Sn whiskers. However, the interstitial Mg form may become a potential source for compressive stresses in the solder, thus accelerating the Sn whisker growth. Additionally, the formation and growth of Sn whiskers may be restricted if diffusion of the Sn atoms is blocked, *e.g.*, by adding barrier atoms or forming lamellar structures in the solders. Our results may help to quickly determine whether a particular solder exhibits a propensity to form Sn whiskers, and they provide scientific basis for determining the Sn whisker risks.

## Methods

**Figure 7:**
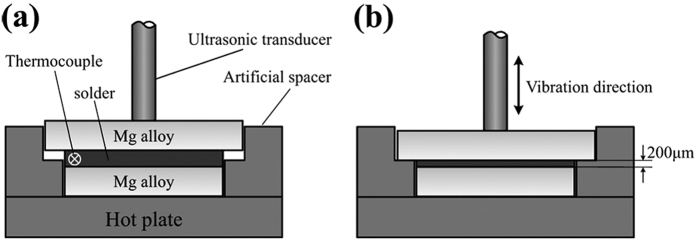
Schematic of the Mg/Sn-based solder/Mg joint (**a**) before and (**b**) after fabrication using our homemade UAS system.

For the experiments, a homemade UAS system equipped with an ultrasonic unit and a heating unit was used to fabricate the Mg/Sn-based solder/Mg sandwich joints (Fig. 7). The Mg sheets were cut into 20 × 10 × 3 or 10 × 10 × 3 mm^3^ pieces, and five compositions of the Sn-based solders (*i.e.*, Sn, SnAg_3.5_, SnCu_0.7_, SnPb_37_ and SnZn_9_) were cut into 10 × 10 × 0.3 mm^3^ pieces. The pieces were then positioned in an overlapping configuration with a faying area of 10 × 10 mm^2^ and a filler spacing of 200 μm created by two artificial spacers. When the solder was heated to 250 °C for 10 s via the heating unit, an ultrasonic vibration was applied for 3–6 s by the ultrasonic unit. The solder temperature was monitored by an inserted K-type thermocouple, and the ultrasonic power, vibration frequency and pressure were fixed at 240 W, 28 kHz, and 0.2 MPa, respectively. Subsequently, the heating and ultrasonic units were stopped, and the resultant sample was cooled to room temperature in air and polished in the cross-sectional direction. Finally, the as-fabricated joints were aged at 25 °C for 0–7 d and then characterized by scanning electron microscopy (SEM, S-4800, Hitachi, Japan) at an accelerating voltage of 15 kV, auger electron spectroscopy (AES, PHI-700, Ulvac-Phi, Japan) at a primary energy of 5 keV, and transmission electron microscopy (TEM, Tecnai G2 F20, Philips, Holland) with a 200 kV accelerating voltage.

**Figure 8:**
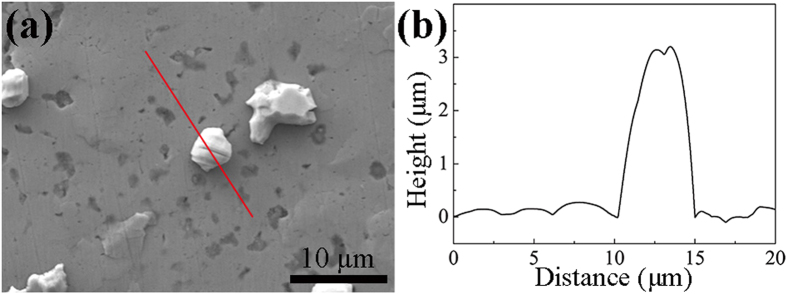
Measurement of the growth length of a Sn whisker: (**a**) SEM image and (**b**) the whisker surface profile via the step-height measurement.

Moreover, a surface profilometer (Alpha-step 500, Tencor, USA) was used to quickly obtain the growth lengths of the short Sn whiskers on the solder surface through a step-height measurement, and the accurate height of a Sn whisker was measured as shown in Fig. 8. The lengths of the long Sn whiskers were measured using the specimen tilting method (for more details, see Section D of the Supplementary material). Furthermore, a differential scanning calorimeter (DSC, STA-449, Netzsch, Germany) was used to determine the formation and growth mechanisms of the Sn whiskers. The test, involving ~30 mg of a particular solder derived from a Mg/Sn/Mg joint after UAS at 250 °C for 6 s, was performed under Ar atmosphere and cycled twice from 25–250 °C at heating/cooling rates of 1 °C/min. Note that the solder was obtained near the Mg sheet by blade cutting. To study the influence of Zn addition on the formation and growth of Sn whiskers, four alloy ingots with compositions of SnCu_0.7_Zn_1_, SnCu_0.7_Zn_3_, SnCu_0.7_Zn_5_, and SnCu_0.7_ Zn_7_ in wt.% were prepared using a melting furnace under Ar atmosphere at 300 °C for 10 h. The ingots were then cut into small pieces (10 × 10 × 0.3 mm^3^), and the pieces were further subjected by the aforementioned fabrication-analysis procedures.

In addition, to identify the influence of the Sn whisker growth on the mechanical properties of the solder matrixes, two Mg/SnCu_0.7_/Mg joints after UAS at 250 °C for 6 s were prepared. One joint was aged at 25 °C for 0–7 d and the other was aged at 150 °C for 0–7 d. Meanwhile, the nanoindentation (UBI, Hysitron, USA) tests were applied to measure the Young’s modulus and hardness of the solder matrixes at each selected time point. Note that the loading force was applied at a constant displacement rate of 10 nm/s until the indentation depth of 100 nm was reached; the dwelling time was 10 s.

## Additional Information

**How to cite this article**: Li, M. Y. *et al.* Fast formation and growth of high-density Sn whiskers in Mg/Sn-based solder/Mg joints by ultrasonic-assisted soldering: Phenomena, mechanism and prevention. *Sci. Rep.* **6**, 27522; doi: 10.1038/srep27522 (2016).

## Supplementary Material

Supplementary Information

## Figures and Tables

**Table 1:** Average growth length of Sn whiskers (*l*, μm), formation densities of Sn whiskers per unit area (ρ, × 10^8^ number/m^2^) and interfacial length (*L*, μm) determined from Fig. 3.

Solder	SnAg_3.5_		SnCu_0.7_		SnPb_37_		SnZn_9_	
Aging time	0 d	7 d	0 d	7 d	0 d	7 d	0 d	7 d
*l*	1.84	7.28	2.43	7.5	0.63	1.07	0.41	0.72
*ρ*	67.2	70.8	60.2	63.2	131	163	3.54	7.08
Δ*V*_*S*_ = *(ρ*_7*•*_*l*_7_* − ρ*_0*•*_*l*_0_)	391.776		327.714		91.88		3.505	
*L*	862.288		858.520		4380.272		1538.859	
*E*_*g*_	High-risk		High-risk		Low-risk		Ultralow-risk	
*E*_*f*_	Moderate-risk		Moderate-risk		High-risk		Ultralow-risk	

In addition, the evaluation of the risk of growing Sn whiskers (*E*_*g*_) and the evaluation of the risk of forming Sn whiskers (*E*_*f*_) in various solders are also provided.
