# Supplementary material for: Fast formation and growth of high-density Sn whiskers in Mg/Sn-based solder/Mg joints by ultrasonic-assisted soldering: Phenomena, mechanism and prevention
Source: Sci Rep. 2016 Jun 8;6:27522. doi: 10.1038/srep27522 (PMC4897630; doi:10.1038/srep27522)
Supplement: Supplementary Information [file srep27522-s1.doc]

**Supplementary material**

**Fast formation and growth of high-density Sn whiskers in Mg/Sn-based solder/Mg joints by ultrasonic-assisted soldering: Phenomena, mechanism and prevention**

***M.Y. Li ^1^, ^*^, H.F. Yang^1^, Z.H. Zhang ^2, #^, J.H. Gu ^1^ & S.H. Yang ^3^***

*^1^State Key Laboratory of Advanced Welding and Joining, Harbin Institute of Technology, Harbin, 150001, China*

*^2^Fujian Key Laboratory of Advanced Materials, Department of Materials Science and Engineering, College of materials, Xiamen University, Xiamen, 361005, China*

*^3^Shanghai Aerospace Equipments Manufacturer, Shanghai, 200245, China*

1. **Identification of the microstructures beneath the Sn whisker**

As shown in Fig. S1a, one Sn whisker, obtained from an as-fabricated Mg/Sn/Mg joint subjected to UAS at 250 °C for 6 s, was cut by the focused-ion beam (FIB, FEI Helios NanoLab 600i) technology. The length of this whisker was close to 5 μm, and its head deviated rightward. More importantly, the root of this whisker was located close to a block-like particle. Based on the EDS analyses in Figs. S1b, the block-like particle (the green region) has a stoichiometry close to Mg_2_Sn, and the red and yellow regions have a stoichiometry close to Sn. Furthermore, the selected area electron diffraction (SAED) pattern of the green region (the white dotted line in Fig. S1b) is recorded in Fig. S1c. The pattern belonged to the [01$\overline{1}$] zone axis of the Mg_2_Sn phase, and the clear diffraction rings at the (211) and (311) surfaces demonstrated that this block-like Mg_2_Sn particle should be polycrystalline. Therefore, we can safely conclude that the roots of the Sn whiskers should be located at the Mg_2_Sn/Sn interface.

1. **Digital imaging method for measuring the Mg concentrations in the solder**

Both the ideal and calculated concentrations of Mg atoms in the solder were calculated using a digital imaging method. Before the calculation, each SEM image was selected using proprietary software, copied, and then imported into the image processing software Adobe Photoshop. Subsequently, the selected regions (*i.e.*, the solder, Mg sheets and the Mg_2_Sn phase) were separated using the Magic Wand tool and the Select Similar command. Quantitative measurements (*e.g.*, the area and perimeter) were obtained by counting the pixels that were inside each region. Finally, the Mg concentration in the solder was calculated using the following equations.

***The ideal concentration of Mg atoms in the solder*** was calculated based on the dissolution of Mg sheets into the solder. As previously mentioned, the average width of the soldering seam after UAS was always wider than the width limited by the artificial spacers; this was observed due to the dissolution. Hence, if the average width of the Mg dissolution is Δ*d*, then the ideal molar concentration of Mg atoms in the solder can be given by the following equation:

$C_{ideal}=\frac{\frac{\Delta d\cdot L\cdot\Delta h\cdot\rho_{Mg}}{M_{Mg}}}{\frac{\Delta d\cdot L\cdot\Delta h\cdot\rho_{Mg}}{M_{Mg}}+\frac{d_{0}\cdot L\cdot\Delta h\cdot\rho_{Sn}}{M_{Sn}}}=\frac{\Delta d\cdot\rho_{Mg}M_{Sn}}{d\cdot\rho_{Mg}M_{Sn}+d_{0}\cdot\rho_{Sn}M_{Mg}}$, (S1)

where Δ*h* and *L* are the depth and length of the soldering seam, respectively; *ρ*_Mg_(= 1.738 g/cm^3^) and *ρ*_Sn_(= 7.365 g/cm^3^) are the densities of the Mg and Sn phases, respectively; *M*_Mg_(= 24.305 g/mol) and *M*_Sn_(= 118.71 g/mol) are the molar masses of the Mg and Sn phases, respectively; and *d*_0_ is the ideal width of the soldering seam.

***The concentration of Mg atoms in the solder*** was measured based on the area of the Mg_2_Sn phase in the solder. If the area of the solder is assumed to be *S_1_* and the area of the Mg_2_Sn phase in the solder is assumed to be *S*_Mg2Sn_, then the calculated molar concentration of the Mg atoms in the solder is given by the following equation:

$\begin{matrix} C_{Calculated}=\frac{\frac{S_{Mg2Sn}\cdot\Delta\text{h}\cdot\rho_{Mg2Sn}}{M_{Mg2Sn}}\times2}{\frac{S_{Mg2Sn}\cdot\Delta\text{h}\cdot\rho_{Mg2Sn}}{M_{Mg2Sn}}\times3+\frac{\left(S_{1}-S_{Mg2Sn}\right)\cdot\Delta h\cdot\rho_{Sn}}{M_{Sn}}} \\ =\frac{2S_{Mg2Sn}\cdot\rho_{Mg2Sn}\cdot M_{Sn}}{3S_{Mg2Sn}\cdot\rho_{Mg2Sn}\cdot M_{Sn}+(S_{1}-S_{Mg2Sn})\cdot\rho_{Sn}\cdot M_{Mg2Sn}} \end{matrix}$, (S2)

where *M*_Mg2Sn_(= 167.32 g/mol) and *ρ*_Mg2Sn_(= 3.53 g/cm^3^) are the molar mass and density of the Mg_2_Sn phase, respectively.

For example, Table S1 shows the calculated data based on the SEM image in Fig. 3a. Because Δ*d* is calculated as 7.89 μm and *d*_0_ is equal to 100 μm, the ideal concentration of Mg in the solder will be 8.34%. Similarly, because *S_1_* = 2.29 × 10^-8^ m^2^ and *S*_Mg2Sn_ = 1.86 × 10^-9^ m^2^, the calculated concentration of Mg in the solder is 5.55%.

1. **Calculation of the strain energy change**

If the atomic concentration of the interstitial Mg in the solder is *c*, then the maximal volume increase in the solder (Δ*v*) through the formation of the Mg_2_Sn particles will be as follows:

$c=\frac{2\cdot\Delta v/(V_{Mg2Sn}-V_{S})}{2\cdot\Delta v/(V_{Mg2Sn}-V_{S})+v_{S}/V_{S}}\xrightarrow{}\frac{\Delta v}{v_{S}}=\frac{1}{2}\cdot\frac{c}{1-c}\cdot\frac{V_{Mg2Sn}-V_{S}}{V_{S}}$, (S3)

where *V*_Mg2Sn_ and *V*_S_ are the molar volumes of the Mg_2_Sn and Sn phases, respectively, and *v_s_* is the solder volume. However, if the bulk modulus is assumed to be the energy required for compressing a globe of volume *v*_s_+*Δv* to *v*_s_, then Δ*u_s_* = (1/2)B(Δ*v/v*_s_)^2^. Therefore, the strain energy change of the solder due to the formation of Mg_2_Sn should be as follows:

$\Delta E_{S}=\Delta u_{s}=\frac{1}{2}B(\frac{\Delta v}{v_{S}})^{2}=\frac{1}{8}B(\frac{c}{1-c})^{2}(\frac{V_{Mg_{2}Sn}-V_{S}}{V_{S}})^{2}$. (S4)

1. **Measurement of the Sn whisker lengths by the specimen tilting method**

For the specimen tilting method (as illustrated in Fig. S2a), we first horizontally rotate the sample stage of the SEM to project the long axis of the whisker (the red straight line of A_1_O) along the *y_1_* axis in the *x*-*y_1_* plane, and then, we record the whisker’s projected length (the blue straight line of B_1_O). Second, we fix the *x-*axis and tilt the sample stage to 30^o^ clockwise. Thus, the *x*-*y_1_* plane is rotated to the *x*-*y_2_* plane, and the points of A_1_ and B_1_ are rotated to A_2_ and B_2_, respectively. Because the *x*-*y_1_* plane is still the observation plane of the SEM, the projected length of the Sn whisker after tilting should be the white straight line of CO (*i.e.*, the projected length of A_2_O in the *x*-*y_1_* plane). Note that both B_1_O and CO can be directly measured from the SEM image. Finally, the following calculations can be performed using Eq. S5 and Eq. S6:

$A_{1}O\cos\alpha=B_{1}O$ (S5)

$A_{2}O\cos(\alpha-30^{o})=CO$ (S6)

where A_1_O=A_2_O is the actual length of the Sn whisker, and *α* is the intersected angle between the long axis of the Sn whisker and the *x*-*y_1_* plane. Accordingly, we have the following equations:

$\alpha=arc\tan(\frac{2CO}{B_{1}O}-\sqrt{3})$ (S7)

$A_{1}O=\frac{B_{1}O}{\cos(arc\tan(\frac{2CO}{B_{1}O}-\sqrt{3}))}$ (S8)

For example, the projected length of a Sn whisker in the *x*-*y_1_* plane (*i.e.*, B_1_O) is 21.8 μm in Fig. S2b, while the projected length of this whisker in the *x*-*y_2_* plane (*i.e.*, CO) is 38.8 μm in Fig. S2c; thus, we obtain *α=*61.3^o^ and A_1_O=45.5 μm. Figure S2d is the cross-sectional image of this whisker obtained by FIB cutting. Apparently, the actual intersecting angle between the Sn whisker and the solder surface is 61.4^o^, which is essentially in agreement with our calculation (61.3^o^) by the specimen tilting method. Therefore, the data of Sn whisker lengths obtained by the specimen tilting method should be accurate in our manuscript.

Table S1 Data calculated based on the SEM image in Fig. 1a

| Image | Original image | Solder image | Mg sheet image | Mg_2_Sn image |
| --- | --- | --- | --- | --- |
|  | 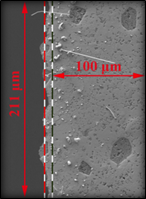 | 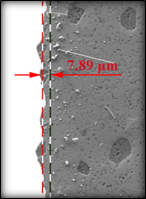 | 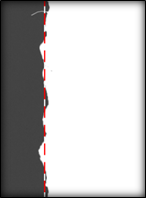 | 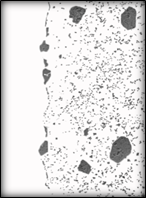 |
| Area (m^2^) | 3.18 × 10^-8^ | 2.29 × 10^-8^ | 8.9 × 10^-9^ | 1.86 × 10^-9^ |

*
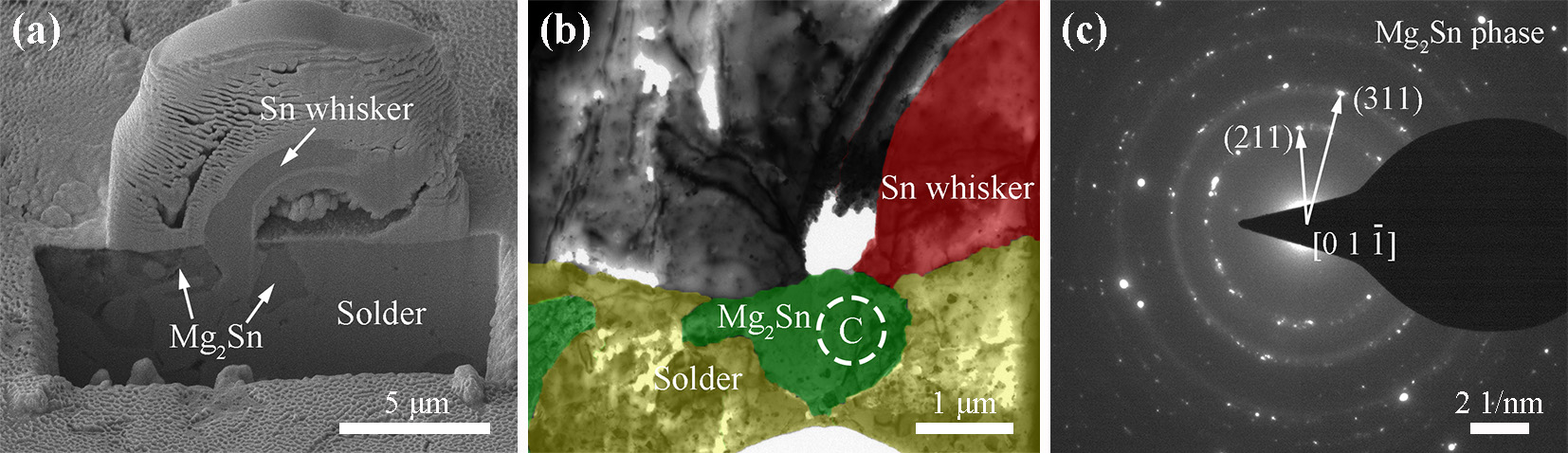
*

Fig. S1 Cross-sectional microstructures beneath one Sn whisker, obtained from an as-fabricated Mg/Sn/Mg joint subjected to UAS at 250 °C for 6 s: (a) SEM image after FIB; (b) low-magnification TEM image, and (c) the SAED image taken from the green region of TEM image.


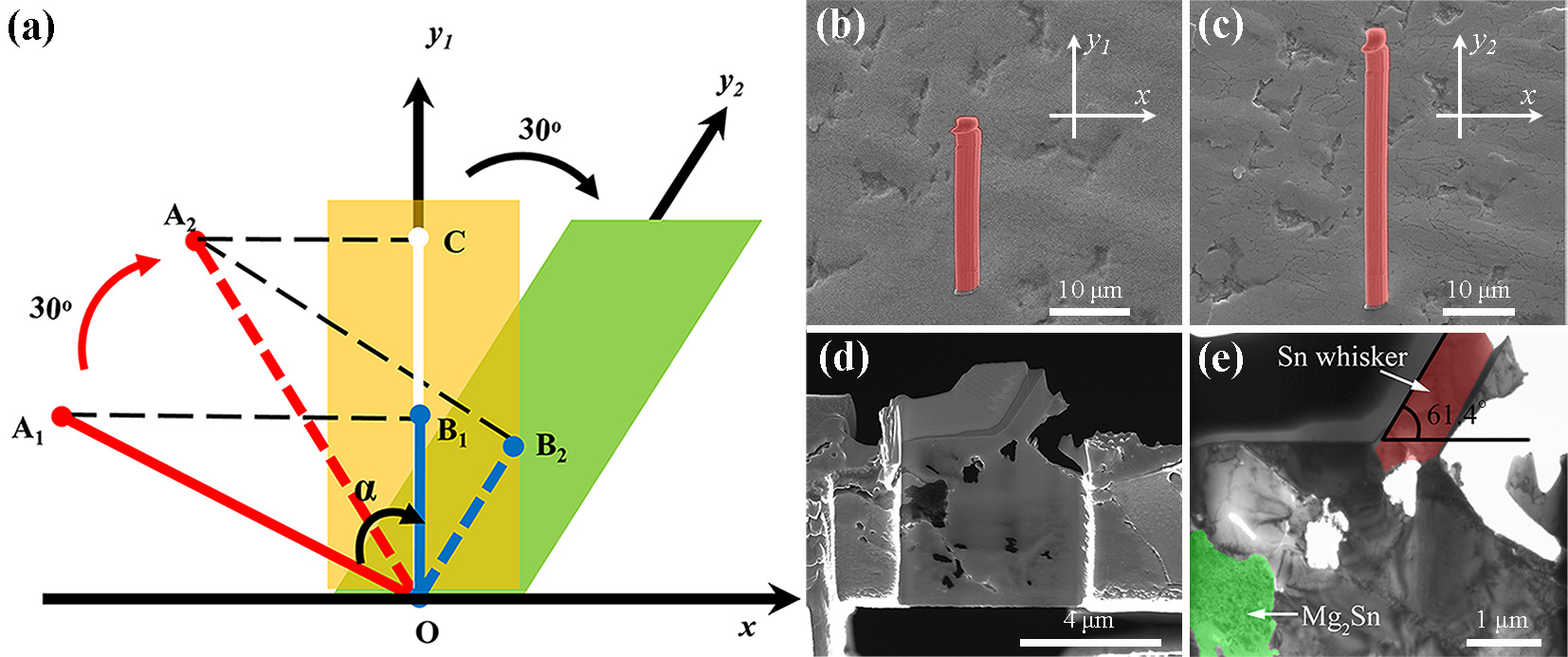


Fig. S2 (a) Sketch map of the measurement of the Sn whisker length using the specimen tilting method. SEM images of a Sn whisker in the top-view direction (b) before tilting and (c) after tilting. TEM images of a Sn whisker in cross-sectional direction (d) at low magnification and (e) at high magnification.
